# Nigericin-induced apoptosis in acute myeloid leukemia via mitochondrial dysfunction and oxidative stress

**DOI:** 10.32604/or.2025.062951

**Published:** 2025-07-18

**Authors:** BHAVYADHARSHINI ARUN, PRARTHANA GOPINATH, ANUP JHA, NISHTHA TRIPATHI, SYED G DASTAGER, SYED K HASAN

**Affiliations:** 1 Hasan Lab, Advanced Centre for Treatment, Research and Education in Cancer (ACTREC), Tata Memorial Centre, Navi, Mumbai, 410210, India; 2 Department of Biotechnology, Indian Institute of Technology Madras (IIT Madras), Chennai, 600036, India; 3 Homi Bhabha National Institute (HBNI), Anushaktinagar, Mumbai, 400094, India; 4 National Collection of Industrial Microorganisms (NCIM), CSIR National Chemical Laboratory, Pune, 411008, India

**Keywords:** Acute myeloid leukemia (AML), Nigericin, Apoptosis, Mitochondrial dysfunction, Antineoplastic agents

## Abstract

**Background:**

Acute Myeloid Leukemia (AML) is a highly aggressive clonal hematological malignancy with limited treatment options. This study aimed to evaluate the therapeutic potential of nigericin, a polyether ionophore derived from *Streptomyces* DASNCL-29, as a mitochondrial-targeted agent for AML treatment.

**Methods:**

Nigericin was isolated from *Streptomyces* DASNCL-29 and characterized via chromatography and NMR. Its cytotoxicity was tested in MOLM13 (sensitive and venetoclax-resistant) and HL60 (sensitive and cytarabine-resistant) cells using the MTT assay. Mitochondrial dysfunction was assessed by measuring reactive oxygen species (ROS), mitochondrial membrane potential (Δψm), and mitochondrial mass. Apoptosis was evaluated with Annexin V/PI assays and immunoblotting, while proteomic analysis was conducted using Liquid Chromatography-Tandem Mass Spectrometry (LC-MS/MS) to identify differentially regulated proteins.

**Results:**

Nigericin demonstrated potent cytotoxicity with IC_50_ values of 57.02 nM in MOLM13-sensitive, 35.29 nM in MOLM13-resistant, 20.49 nM in HL60-sensitive, and 1.197 nM in HL60-cytarabine-resistant cells. Apoptosis was confirmed by Annexin V/PI staining and caspase-3/PARP cleavage, along with MCL-1 downregulation. Mitochondrial dysfunction was evident from increased ROS, reduced Δψm, and decreased mitochondrial mass. Proteomic profiling identified 264 dysregulated proteins, including a 3.8-fold upregulation of Succinate Dehydrogenase [Ubiquinone] Flavoprotein Subunit A (SDHA).

**Conclusion:**

Nigericin induces apoptosis in AML cells by disrupting mitochondrial function and enhancing oxidative stress. Its nanomolar potency highlights the need for further mechanistic studies and *in vivo* evaluations to explore its potential in AML treatment.

## Introduction

Acute Myeloid Leukemia (AML) is a rapidly progressing myeloid neoplasm that originates in the bone marrow, characterized by the clonal expansion of immature myeloid-derived cells known as blasts [1,2]. Recent advancements have significantly improved the efficacy of mitochondrial-targeted therapies in treating AML [3,4]. AML cells are characterized by excessive accumulation of reactive oxygen species (ROS) due to increased oxidative stress and weakened antioxidant defenses [5,6]. A hallmark of AML, particularly in relapsed/refractory cases, is its metabolic dependency on oxidative phosphorylation (OXPHOS) for survival, a feature that distinguishes it from many other cancers and amplifies its sensitivity to mitochondrial perturbations [7,8]. This OXPHOS-driven redox imbalance not only promotes genomic instability but also creates a therapeutic vulnerability, where further ROS elevation can overwhelm AML’s antioxidant buffering capacity, selectively triggering apoptosis in leukemic cells while sparing normal hematopoietic cells with lower baseline ROS [9,10]. This strategy is particularly relevant for eliminating leukemic stem cells (LSCs), which, although inherently resistant to standard therapies due to their quiescent nature, remain vulnerable to disruptions in redox homeostasis [11–13]. Critically, LSCs in refractory AML retain OXPHOS dependency, as demonstrated by the persistence of oxidative metabolism in venetoclax-resistant clones [14,15]. This metabolic reliance renders them susceptible to mitochondrial-targeted agents, bypassing resistance mechanisms to conventional therapies [16–18]. Furthermore, the inherent heterogeneity of AML complicates the identification of universally effective therapies, as different genetic mutations and resistance mechanisms can lead to varied responses among patients [19–21]. In light of these challenges, drug repurposing has emerged as a promising strategy for developing new treatments for AML [22].

Nigericin is a polyether ionophore originally discovered as an antibiotic derived from the bacterium *Streptomyces hygroscopicus* [23]. It possesses unique chemical properties that enable it to facilitate the transport of cations, particularly potassium (K^+^), hydrogen (H^+^), across cellular membranes, thereby influencing ion homeostasis and pH regulation within cells [24]. By collapsing mitochondrial ion gradients, nigericin disrupts OXPHOS, exacerbates ROS accumulation, and impairs pH-dependent antioxidant defences [25,26]. Therefore, the study hypothesizes that AML’s OXPHOS addiction lowers the threshold for ionophore-mediated cytotoxicity compared to non-malignant cells, as shown by the selective targeting of LSCs by other ionophores like salinomycin in preclinical models [27]. This mechanism is uniquely suited to target AML’s metabolic vulnerabilities [28–30]. The study proposes that nigericin, like salinomycin in AML [31] and UM4118 in SF3B1-mutant leukemia [32] could exploit ionophoric properties to target AML. Specifically, it has the ability to modulate ion flux, inhibit the Wnt/β-catenin and SRC/STAT3 pathways, and induce pyroptosis [24,33,34]. Combined with mitochondrial destabilization, it may eradicate apoptosis-resistant clones.

This study explored the effects of nigericin, a compound derived from a novel *Streptomyces* strain DASNCL-29, on MOLM13 Parental and MOLM13 Venetoclax-resistant cells. The venetoclax-resistant model was chosen since resistance to BCL-2 inhibition is mechanistically linked to retained OXPHOS dependency, providing a rationale for nigericin’s efficacy in this context [14,15]. This study’s findings suggest that nigericin induces apoptosis by disrupting mitochondrial dynamics, warranting further investigation to establish its potential as a therapeutic candidate for AML.

## Materials & Methods

### Isolation and characterisation of nigericin from a novel Streptomyces strain

This study used an earlier characterized nigericin metabolite from a novel indigenous species of *Streptomyces* strain DASNCL-29, which was isolated from a plant-associated soil sample collected from Unkeshwar, Maharashtra, India. A seven-day log-phase culture of DASNCL-29 was used for fermentation to produce the bioactive compound nigericin. The fermentation was carried out in a 10-L lab-scale fermenter (Eppendorf BioFlow^®^ CelliGen^®^ 115, Hamburg, Germany). The harvested fermentation broth was used to extract the metabolite, and purification was carried out by gradient column chromatography and High-Performance Liquid Chromatography (HPLC, UltiMate 3000, ThermoFisher Scientific, Waltham, MA, USA). Further, structural elucidation was done with standard Nuclear Magnetic Resonance techniques (NMR) (Bruker Avance III HD 400 MHz spectrometer, Bruker BioSpin GmbH, Silberstreifen 4, 76287, Rheinstetten, Germany). The structural characteristics of purified nigericin were analyzed using spectral techniques, including Heteronuclear Multiple Bond Correlation (HMBC), Nuclear Overhauser Effect Spectroscopy (NOESY), and Correlation Spectroscopy (COSY) [35].

### Anticancer potential of nigericin

The anticancer effects of nigericin were evaluated against 17 cancer cell lines including U-937 (human myeloid leukemia), MDA-MB-468 (human breast cancer), HOP-62 (human lung cancer), SCC-40 (human oral squamous cell carcinoma), HL-60 (human leukemia), HT-29 (human colon cancer), COLO-205 (human colon cancer), HeLa (human cervical cancer), JURKAT (human T-cell leukemia), SiHa (human cervical cancer), DU-145 (human prostate cancer), Hep-G2 (human hepatocellular carcinoma), K-562 (human chronic myelogenous leukemia), MCF-7 (human breast cancer), A-549 (human lung carcinoma), PC-3 (human prostate cancer), and MDA-MB-231 (human breast cancer) with Vero cell line as a control. (Advanced Centre for Treatment, Research and Education in Cancer (ACTREC), Mumbai, India) using the sulforhodamine B (SRB) (0.4 % (w/v) in 1 % acetic acid) (Cat No. 230162, Sigma-Aldrich, St. Louis, MO, USA) assay. All the cell lines used in this experiment were authenticated using short tandem repeat (STR) profiling, and their mycoplasma contamination status was routinely monitored to ensure experimental validity. Cells were seeded in 96-well plates (5000 cells/well) in RPMI 1640 medium supplemented with 10% fetal bovine serum and 2 mM L-glutamine (SLM-240, Sigma-Aldrich), followed by 24 h incubation at 37°C and 5% CO_2_. Nigericin and Doxorubicin, dissolved in DMSO (10^−2^ M stock), were serially diluted to final concentrations of 100, 10, 1.0, 0.10 µM. After 48 h of treatment, cell viability was assessed using the SRB.

Percentage Growth was calculated using the equation

$$Percentage\;Growth\left(\%\right)=\left[\frac{\left(\mathrm{Ti}-\mathrm{Tz}\right)}{C-\mathrm{Tz}}\right]\times 100$$

where *C* represents the control absorbance, *Ti* the test sample absorbance, and *Tz* the baseline (time zero) absorbance.

Synthetic compounds showing Growth inhibition of 50% (GI_50_) (calculated from [(Ti-Tz)/(C-Tz)] × 100 = 50) ≤10 μg/mL were considered to be exhibiting significant anticancer activity.

### AML cells and culture conditions

The MOLM13 paired sensitive and venetoclax-resistant cell lines were procured from the European Institute of Oncology, Milan, Italy, and HL60 paired sensitive and Cytarabine-resistant cells from the University Hospital, Leipzig, Germany. The authentication of cell lines was confirmed through STR profiling in February 2024, with concurrent verification of mycoplasma contamination-free status. The procured cells were maintained in RPMI medium (Gibco, Grand Island, CA, USA) supplemented with 10% fetal bovine serum (Gibco) and penicillin-streptomycin (Himedia, Mumbai, India). The cultures were incubated under standard conditions, and subculturing was performed every three to five days once the cells reached 60%–80% confluency.

### Cytotoxicity analysis

1 × 10^4^ MOLM13 sensitive and venetoclax-resistant and 1 × 10^4^ HL60 sensitive and cytarabine-resistant cells were seeded per well in a 96-well plate, with different concentrations (0, 12.5, 25, 50, 100, and 200 nM) of nigericin. The plates were incubated for 48 h under standard conditions. After the incubation period, 3-(4,5-dimethylthiazol-2-yl)-2,5-diphenyltetrazolium bromide (MTT) reagent (Himedia) at 1 mg/mL. The plates were further incubated for an additional 5 h to allow formazan crystal formation. The crystals were solubilized using a solubilizing buffer (Isopropanol, Triton X-100, and concentrated HCl), and the plate was left overnight at 37°C to ensure complete solubilization of the crystals. Absorbance was recorded at 570 nm using a microplate reader (BioTek, Cytation 5, Winooski, VT, USA). The absorbance readings in each treatment group were normalized to the untreated group to acquire percentage viabilities. The concentrations were transformed into a logarithmic scale, and a non-linear regression curve for log (inhibitor) vs. normalized response-variable slope was used to calculate the IC_50._

### Cell proliferation assay

MOLM13 sensitive cells were labelled with the membrane-permeable fluorescent dye Carboxyfluorescein Diacetate Succinimidyl Ester (CFSE) (Cat No. C34554, Invitrogen, ThermoFisher Scientific) prior to nigericin treatment. CFSE (2 µM in DMSO) was freshly diluted 100-fold in 1× PBS (pH 7.4), and the cells were incubated with the dye for 10 min in the dark at room temperature. Complete RPMI medium was added to terminate the reaction, followed by incubation at 37°C for 10 min. Afterward, 1.0 × 10^6^ cells/mL in fresh RPMI medium were harvested and seeded. After a 24-h incubation, the cells were washed with PBS, resuspended, and analysed by flow cytometry using the Attune NxT Flow Cytometer (ThermoFisher Scientific).

### Assessment of apoptosis by flow cytometry

1 × 10^6^ MOLM13 sensitive (with or without 5 µM. MitoTempo (MedChemExpress, Cat No. HY-112879, Monmouth Junction, NJ, USA) pretreatment) and MOLM13 and venetoclax-resistant cells were seeded in a 6-well plate with 5 mL of complete RPMI and treated with different concentrations of nigericin. After a 24-h incubation, the cells were harvested, washed with 1× PBS (pH 7.4) and resuspended in 1X annexin binding buffer and stained with Annexin V conjugated with Alexa Fluor 488 and Propidium Iodide (Cat No. V13241, Invitrogen, Thermo Fisher Scientific) according to the manufacturer’s instructions and acquired on an Attune NxT Flow Cytometer.

### Immunoblotting

MOLM13 sensitive and venetoclax-resistant cells were treated with nigericin (20, 40, and 80 nM) for 24 h, then harvested and lysed using RIPA buffer (Cat No. R0278, Sigma-Aldrich) on ice for 45 min. Protein concentration was measured using the Bradford reagent (Himedia) according to the manufacturer’s instructions. A total of 40 µg of protein was loaded onto 10% or 12% SDS-PAGE gels, resolved, and transferred to nitrocellulose membranes (Cytiva, Marlborough, MA, USA). The membranes were blocked with 5% skimmed milk (Himedia) or BSA (Himedia), incubated overnight with primary antibodies including Succinate Dehydrogenase [Ubiquinone] Flavoprotein Subunit A (SDHA) (Cat No. PA5-27482, dilution 1:1000, ThermoFisher Scientific), Full length PARP (Cat No. 9532, dilution 1:1000, Cell Signaling Technology, Danvers, MA, USA), Cleaved PARP (Cat No. 32563, dilution 1:1000, Cell Signaling Technology), Cleaved Caspase-3 (Cat No. 9664, dilution 1:1000, Cell Signaling Technology), γH2AX (Cat No. 2577, dilution 1:1000, Cell Signaling Technology), MCL-1 (Cat No. 94296, dilution 1:1000, Cell Signaling Technology) at 4°C overnight, and then incubated with HRP-conjugated secondary antibody (Cat No. 7074, dilution 1:3000, Cell Signaling Technology) at room temperature for 1 h. Unbound primary and secondary antibodies were washed with TBS-T buffer (1M Tris pH 8, 2.5M NaCl, Tween 20, and double-distilled water) thrice for 15 min. After washing, protein bands were visualized using Cytiva ECL select™ western blotting detection reagent (Cat. No. RPN2235) and Bio-Rad Chemidoc system, California, USA (12003153).

### Proteomic profiling

Approximately 2 × 10^6^ MOLM13 sensitive cells, both untreated and 100 nM nigericin-treated, were used for protein isolation. The cells were lysed in a native buffer containing 20 mM Tris, 150 mM NaCl, 10% glycerol, and 0.1% NP-40, adjusted to pH 7.5. Following lysis, proteins were subjected to reduction with 10 mM Dithiothreitol (DTT) and alkylation using 20 mM iodoacetamide (IAA). Subsequent to reduction and alkylation, the proteins were digested overnight with trypsin at 37°C. The completeness of digestion was verified by SDS-PAGE. The digested samples were then cleaned using C18 ZipTips (Cat No. ZTC18S096, Merck Millipore, Burlington, MA, USA) prior to analysis by Liquid Chromatography Tandem Mass Spectrometry (LC-MS/MS) (nLc-ESI-Q-TOF, Triple TOF 5600 plus, SCIEX, Framingham, MA, USA).

### Measurement of mitochondrial ROS

Mitochondrial ROS levels were quantified using the MitoSOX™ Red probe (Cat No. M36008 ThermoFisher Scientific). MOLM13 sensitive and venetoclax-resistant cells (1 × 10^6^ cells/well) were treated with 20, 40, and 80 nM concentrations of nigericin or left untreated as a control, in a 6-well plate. After 24 h of incubation, the cells were harvested, washed with PBS and stained with 5 μM of MitoSOX Red for 15 min in the dark. The cells were then washed with PBS and analysed using flow cytometry.

### Evaluation of mitochondrial membrane potential

1 × 10^6^ MOLM13 sensitive and venetoclax-resistant cells were seeded in a 6-well plate. After 24 h of incubation, the cells were harvested, washed with 1× PBS (pH 7.4), and stained with JC-1 probe (Cat No. HY-15534, MedChemExpress) according to the manufacturer’s instructions. The cells were then washed with 1× PBS (pH 7.4) and analysed using an Attune NxT flow cytometer.

### Quantification of mitochondrial mass

1 × 10^6^ MOLM13 sensitive cells were seeded and treated with nigericin for 24 h. Post-treatment, the cells were collected and washed with 1× PBS (pH 7.4). Subsequently, the cells were incubated with 50 nM Mitotracker Green (Cat No. M6514, Invitrogen, ThermoFisher Scientific) at 37°C for 30 min. Following this staining, the cells were washed with PBS and then stained with Hoechst 33342 (Cat No. 62249, Invitrogen, ThermoFisher Scientific) for 10 min at 37°C. Finally, the cells were transferred to poly-L-lysine-coated glass-bottom dishes and visualized using a Nikon AX Eclipse Ti2 confocal microscope (New York, NY, USA). The mean fluorescence intensity of at least 30 cells per concentration was measured using the Image J version 1.54 g software, National Institutes of Health (Bethesda, MD, USA).

### Statistical analysis

The data are represented as mean ± standard deviation (SD) unless otherwise specified. Statistical analyses were conducted using GraphPad Prism (version 8.4.2 (679), 8 April 2020, San Diego, CA, USA). An unpaired Student’s *t-*test was employed to evaluate differences in cell apoptosis, proliferation, mitochondrial ROS production, mitochondrial membrane potential, and mitochondrial mass. For cell viability assessments, non-linear regression analysis was applied, while simple linear regression was used for the Bradford assay. A significance level of *p* < 0.05 was considered statistically significant.

## Results

### Isolation and structural analysis of bioactive metabolites produced by streptomyces DASNCL-29

Submerged fermentation of *Streptomyces* strain DASNCL-29 yielded 25.0 g of a pure white crystalline compound from 75.0 g of crude extract, which translates to a 33% (w/w) yield. Further, the structural characteristics analysis via the mass spectrum of nigericin from strain DASNCL-29 supported its structural integrity, in comparison with the standard nigericin.

### Anticancer potential of nigericin

**Figure A1:**
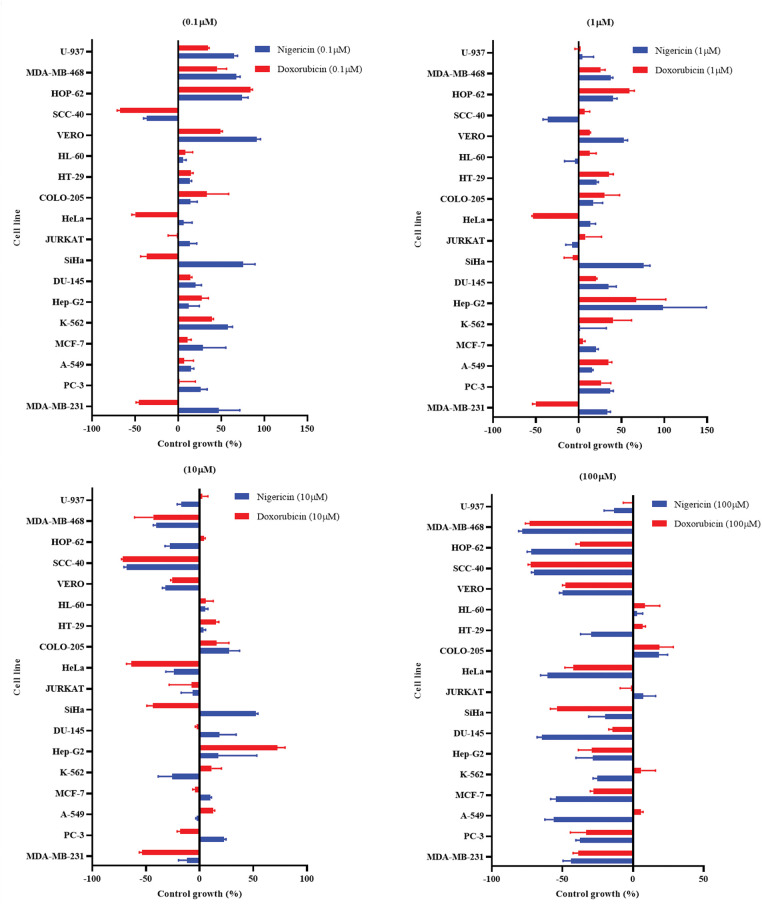
Percentage growth of different cell lines at different drug concentrations (100, 10, 1, and 0.1 µM) of Nigericin and Doxorubicin.

**Figure A2:**
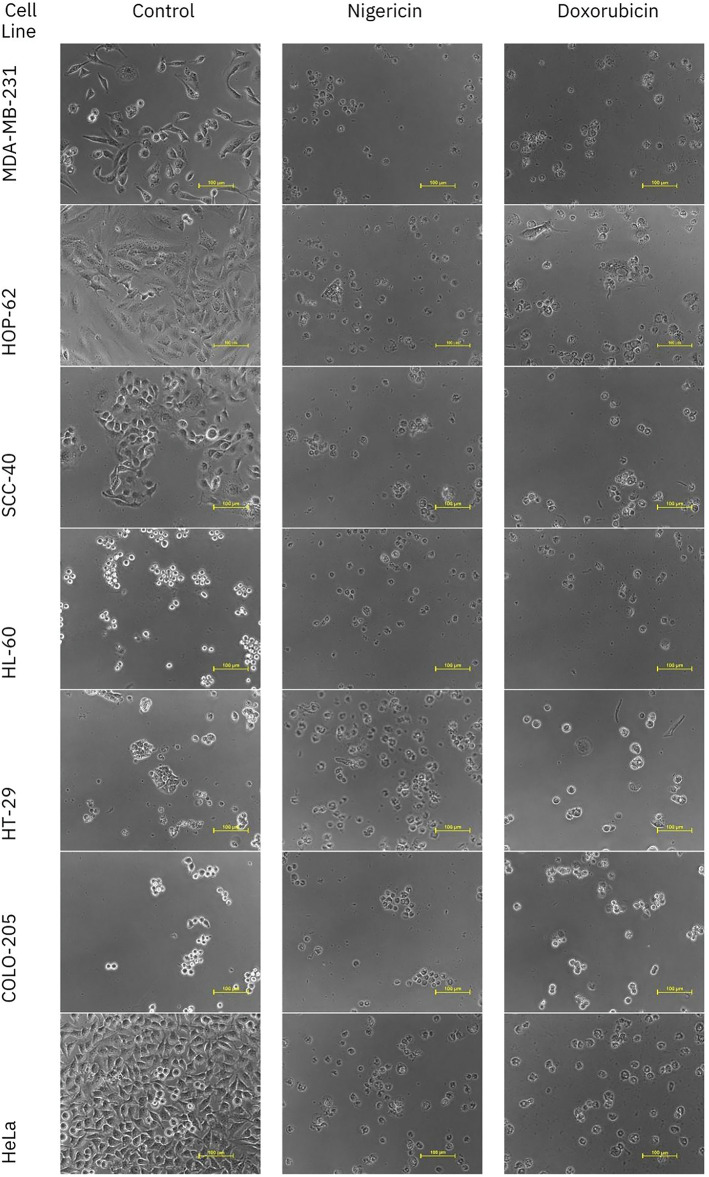

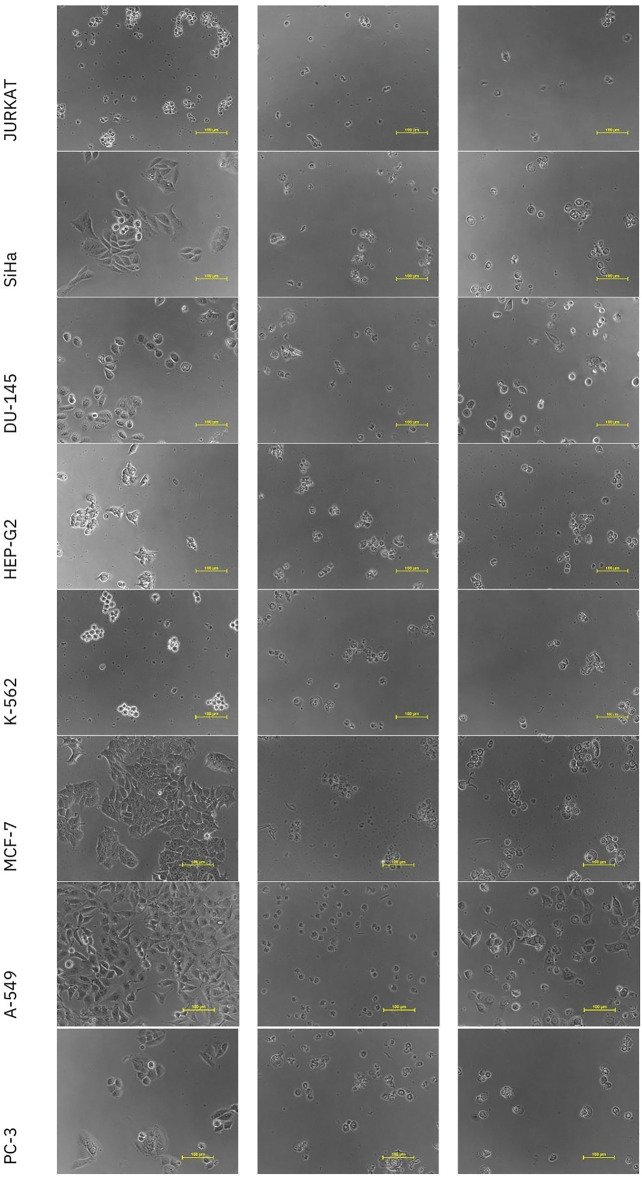
Representative images of *in vitro* anticancer activity of Nigericin and Doxorubicin against different cell lines.

The *in vitro* cytotoxicity of nigericin was evaluated using the sulforhodamine B assay across seventeen human cancer cell lines representing various histological origins. Comprehensive anticancer screening data are presented in Fig. A1. The anticancer potential of nigericin, including its effects on AML cells, was investigated (Table A1). Notably, nigericin induced morphological changes, including cell shrinkage and detachment, as illustrated in Fig. A2, indicative of cell death. These findings emphasize nigericin’s ability to disrupt cellular viability in AML.

### Nigericin inhibits AML cell survival and proliferation in vitro

**Figure 1:**
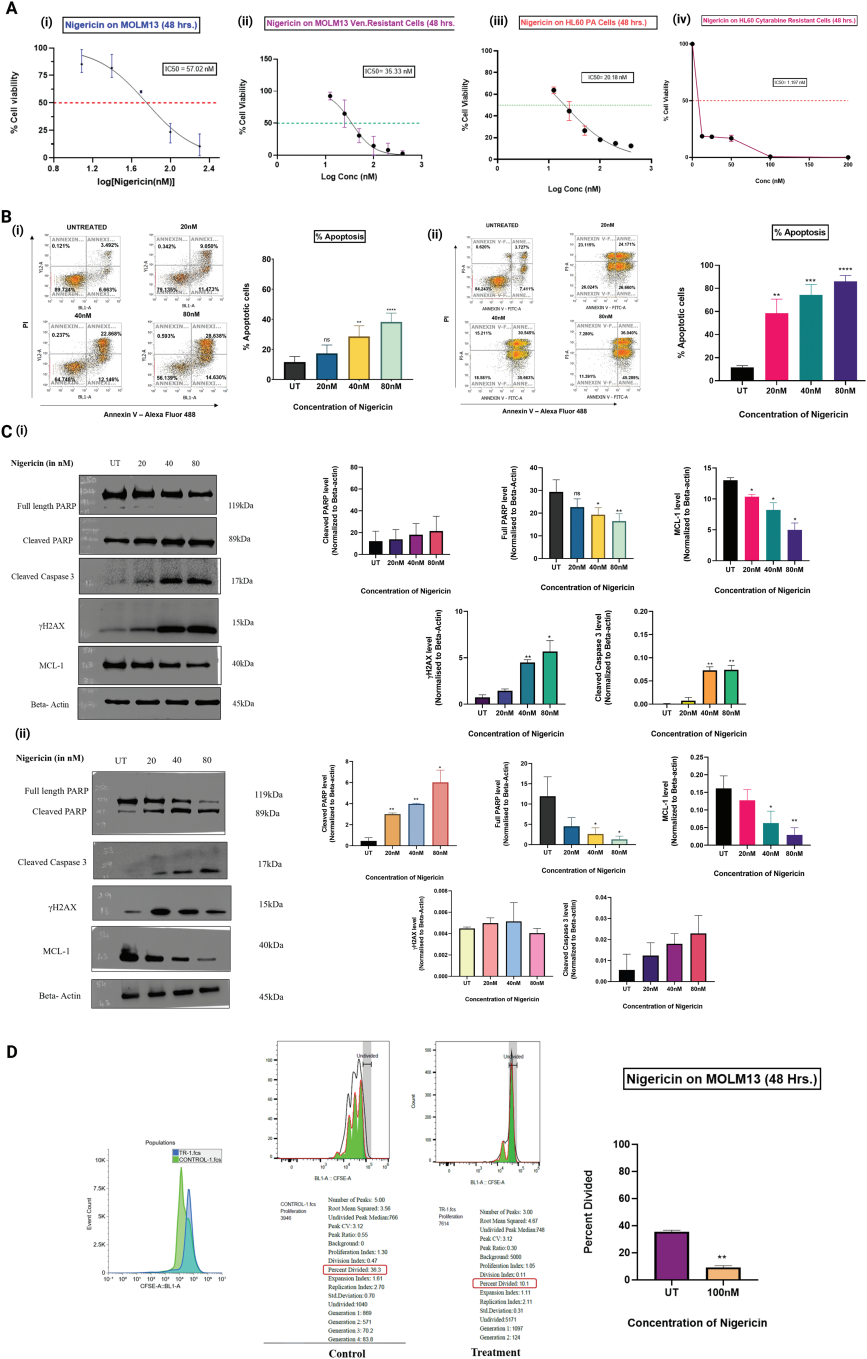
Nigericin decreases cell viability, inhibits proliferation, and induces apoptosis in AML cells. (A) Dose-response curves show the effect of nigericin on cell viability after 48 h in MOLM13 sensitive (IC_50_ = 57.02 nM), venetoclax-resistant MOLM13 (IC_50_ = 35.33 nM), HL60 sensitive (IC_50_ = 20.18 nM), and cytarabine-resistant HL60 (IC_50_ = 1.197 nM) cells. Data represents Mean ± SD (n = 3). (B) The apoptotic levels of nigericin-treated (i) MOLM13 sensitive cells and (ii) MOLM13 venetoclax-resistant cells were evaluated using the Annexin V-Alexa Fluor 488/Propidium Iodide assay. The values were analysed using an unpaired Student’s *t*-test. Data are expressed as mean ± SD (**p < 0.01, ***p < 0.001, ****p < 0.0001, ns, not significant) (n = 3). (C) Immunoblotting of apoptosis-related proteins and γH2AX, using actin as the loading control in (i) MOLM13 sensitive cells and (ii) MOLM13 venetoclax-resistant cells (*p < 0.05, **p < 0.01) (n = 3). (D) Proliferation of MOLM13-sensitive cells measured by CFSE dilution assay, comparing control and 100 nM nigericin-treated groups. Data represents Mean ± SD (**p < 0.01) (n = 2).

MOLM13 sensitive and venetoclax-resistant cells, and HL60 sensitive cells and cytarabine-resistant cells were treated with increasing concentrations of nigericin (12.5–200 nM) for 48 h, and an MTT assay was performed. The results indicated that nigericin reduced the viability of the treated AML cells in a dose-dependent manner, with IC_50_ values of 57.02 and 35.29 nM in MOLM13 sensitive and venetoclax-resistant cells and 20.49 and 1.197 nM in HL60 sensitive cells and cytarabine-resistant cells (Fig. 1A). Further, MOLM13-sensitive and Venetoclax-resistant cells were incubated with different concentrations of nigericin for 24 h, and an Annexin V/PI assay was done. Nigericin was found to significantly increase the percentage of apoptotic cells (early and late) in a dose-dependent manner, whereas the percentage of necrotic cells was not significantly altered (Fig. 1B). Additionally, immunoblotting was performed to validate the induction of apoptosis. Upregulation of apoptotic markers, including cleaved PARP, cleaved caspase-3, and subsequent downregulation of full-length PARP and pro-survival protein MCL-1, was observed. Also, DNA double-strand breakage was confirmed by the upregulation of the protein γH2AX with increasing concentrations of nigericin (Fig. 1C). Next, the CFSE assay was done to check the effect of nigericin on AML cell proliferation. MOLM13 sensitive cells were stained with CFSE dye and treated with or without nigericin for 48 h. The results indicated a reduction in the percentage of cells divided in the 100nM nigericin-treated group, and a peak shift was observed in the control group, compared to nigericin-treated MOLM13 sensitive cells, reflecting the reduced proliferation of treated cells (Fig. 1D).

### Nigericin induces differential protein expression profiles

**Figure 2:**
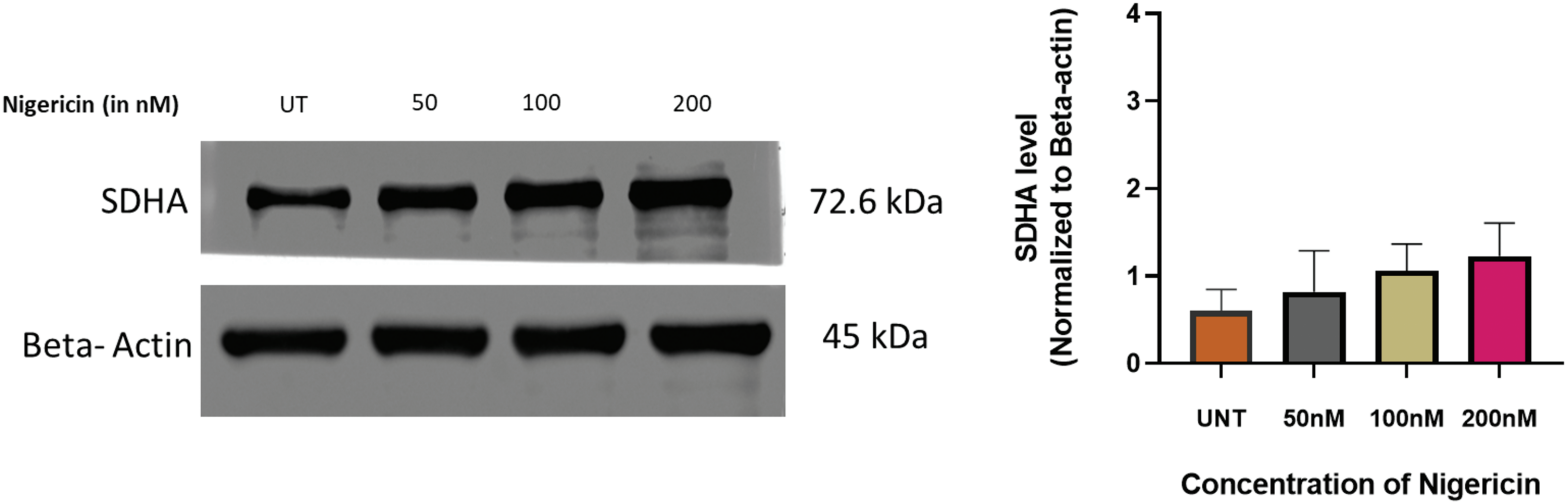
Regulation of SDHA by nigericin. Western blot to detect the expression of SDHA upon nigericin treatment, with β-actin as the internal reference (n = 3).

Proteomic profiling of MOLM13 sensitive cells treated with 100 nM nigericin identified 785 proteins, of which 264 were significantly dysregulated (*p* < 0.05) compared to controls. Among these, 115 proteins were upregulated, with 45 showing a fold change greater than 5.0, while 149 proteins were downregulated, with 39 exhibiting a fold change less than 0.2. Six proteins were further selected based on log-fold changes and statistical significance (Table 1). Among these, Succinate Dehydrogenase [Ubiquinone] Flavoprotein Subunit A (SDHA), an essential enzyme in the mitochondrial respiratory chain, was found to be upregulated and stood out as the protein of interest due to the previously reported role of nigericin to induce mitochondrial dysfunction in breast cancer cells. Further, immunoblotting revealed an upward trend in SDHA expression in nigericin-treated cells, though the increase did not reach statistical significance (Fig. 2).

**Table 1:** List of Proteins shortlisted based on log fold change and statistical significance

Gene symbol	UNIPROT ID	Protein name	Pathway	Log_2_FC
SDHA	P31040	Succinate dehydrogenase [ubiquinone] flavoprotein subunit A, mitochondrial	TCA	3.80
MSH6	P52701	DNA mismatch repair protein	Rb signalling	4.02
MDC1	Q14676	Mediator of DNA damage checkpoint protein 1	Cell cycle progress	6.71
SRSF2	Q01130	Isoform 2 of Serine/arginine-rich splicing factor 2	Spliceosome	−3.52
MNDA	P41218	Myeloid cell nuclear differentiation antigen	Transcription factor	−3.06
CSK2B	P67870	Casein kinase II subunit beta	Wnt signalling	−2.51

### Nigericin significantly alters mitochondrial ROS, mass, and membrane potential

**Figure 3:**
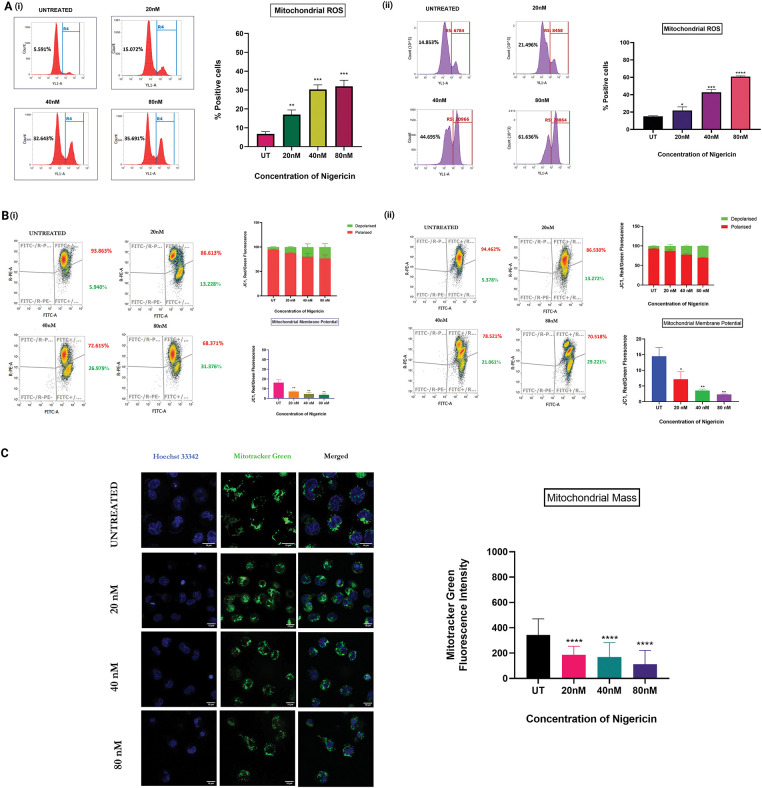
Nigericin alters mitochondrial homeostasis and induces apoptosis via mitochondrial ROS. (A) Increase in mitochondrial ROS levels in a concentration-dependent manner in (i) MOLM13 sensitive cells and (ii) MOLM13 venetoclax-resistant cells (*p < 0.05, **p < 0.01, ***p < 0.001, ****p < 0.0001) (n = 3). (B) Mitochondrial membrane potential (Δψm) measured by JC-1 staining after 24 h nigericin treatment in (i) MOLM13-sensitive and (ii) MOLM13 venetoclax-resistant cells. Data represents Mean ± SD (*p < 0.05, **p < 0.01) (n = 3). (C) A concentration-dependent decrease in mitochondrial mass was observed with increasing nigericin doses in MOLM13 sensitive cells. Statistical significance is indicated as ****p < 0.0001. Data represent mean ± SD (n = 2).

Mitochondrial ROS levels were quantified in MOLM13 sensitive and venetoclax-resistant cells treated with nigericin for 24 h using the MitoSOX Red probe, which selectively detects mitochondrial superoxide production. The results demonstrated a significant increase in mitochondrial ROS, indicating elevated oxidative stress with increasing nigericin concentrations (Fig. 3A). Subsequently, mitochondrial membrane potential (Δψm*)* was assessed in both MOLM13-sensitive and Venetoclax-resistant cells using the JC-1 probe. Under normal conditions, JC-1 accumulates within mitochondria and forms red-fluorescent J-aggregates, indicative of an intact membrane potential. In contrast, a shift to green fluorescence occurs when *Δψm* is disrupted, as JC-1 remains in its monomeric form. Our findings revealed a concentration-dependent shift from red to green fluorescence, confirming that nigericin progressively decreases mitochondrial membrane potential (Fig. 3B). Finally, mitochondrial mass was evaluated in MOLM13 sensitive cells via confocal microscopy using the MitoTracker Green probe. A substantial reduction in mitochondrial mass was observed with increasing nigericin concentrations, further supporting the evidence of mitochondrial dysfunction at higher nigericin concentrations (Fig. 3C).

### Mitochondrial ROS mediates nigericin-induced apoptosis in MOLM13 cells

**Figure 4:**
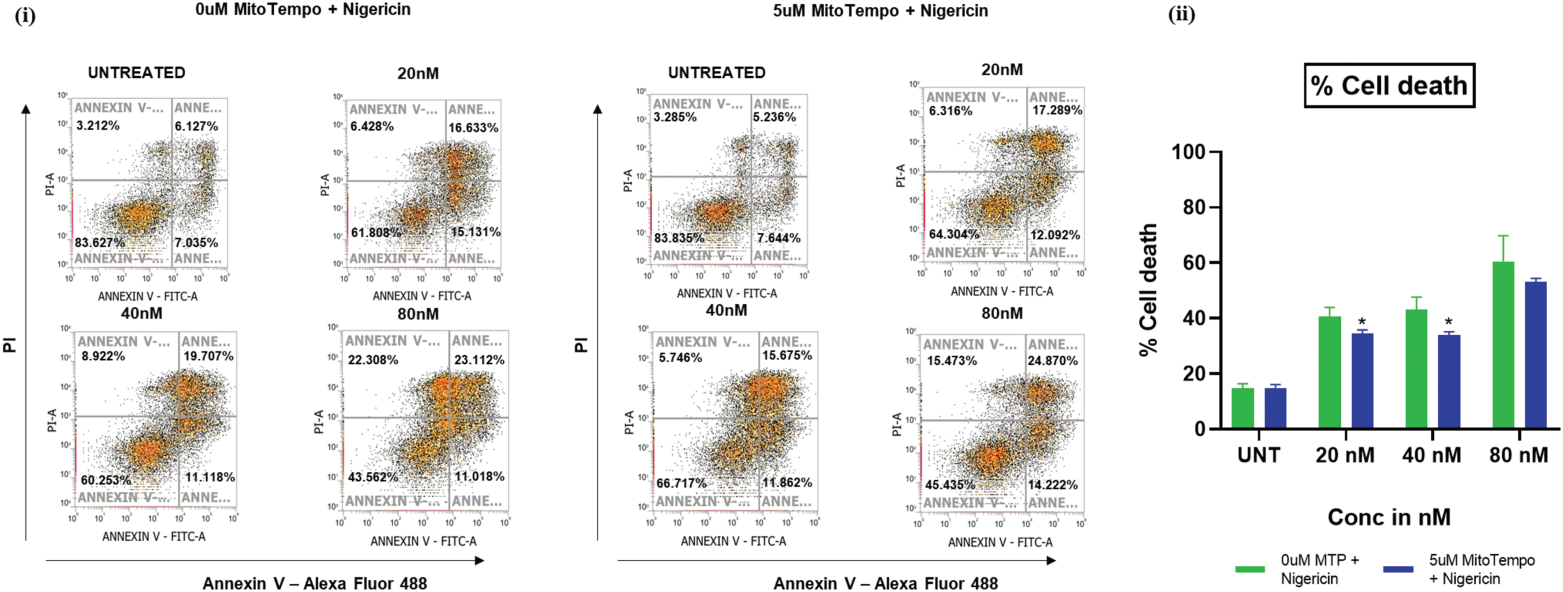
Nigericin induces apoptosis via mitochondrial ROS. Apoptosis analysis by Annexin V-Alexa Fluor 488/PI staining in nigericin-treated MOLM13-sensitive cells ± 5 µM MitoTEMPO pretreatment (*p < 0.05) (n = 3).

An apoptosis assay was performed using Annexin V/PI staining in MOLM13 sensitive cells. They were pretreated with or without 5 µM MitoTempo, a mitochondria-targeted ROS scavenger, followed by treatment with different concentrations of nigericin. Pretreatment with MitoTempo was found to attenuate nigericin-induced cell death, with a marked reduction in the percentage of dead cells compared to cells treated with nigericin alone. This functional linkage demonstrates that mitochondrial ROS elevation is one of the mechanistic drivers of apoptosis induction by nigericin in AML (Fig. 4).

## Discussion

Nigericin’s anticancer effects have been documented in several solid tumors, including lung, breast, and colorectal cancers, where it inhibits key signaling pathways such as Wnt/β-catenin, PI3K/Akt, and MAPK/ERK [22,36]. Our study extends these findings to acute myeloid leukemia (AML), a hematological malignancy characterized by treatment resistance and relapse. Recent studies have highlighted the role of mitochondrial metabolism and oxidative stress in sustaining AML cell survival, particularly in relapsed/refractory cases [8]. Building on this foundation, our findings demonstrate that nigericin, a polyether ionophore derived from *Streptomyces* DASNCL-29, induces apoptosis in AML cells through mitochondrial dysfunction and oxidative stress.

Nigericin’s mechanism aligns with emerging strategies targeting mitochondrial vulnerabilities in AML [3,37,38]. This is consistent with studies showing that LSCs and resistant clones remain dependent on OXPHOS for survival, rendering them susceptible to mitochondrial stressors [13]. For instance, venetoclax-resistant AML cells retain OXPHOS dependency, making them vulnerable to agents that disturb redox balance. The study’s observations that nigericin downregulates MCL-1 and activates caspase-3/PARP cleavage in AML cells further support its role in overcoming apoptosis resistance, a hallmark of refractory AML. Additionally, nigericin may enhance its therapeutic efficacy against AML by potentially lowering intracellular pH and inducing apoptosis through a mechanism similar to that of Sodium-Hydrogen Exchanger 1 (NHE1) inhibitors [39].

Proteomic analysis revealed that nigericin upregulated SDHA, a succinate dehydrogenase (Complex II) subunit, in AML cells (Fig. 2). While SDHA is typically associated with mitochondrial respiration, its overexpression under stress conditions has been linked to ROS amplification and apoptosis in cancer cells [40]. This paradoxical response, an attempt to compensate and restore energy production, may exacerbate oxidative damage, as observed with other ionophores like salinomycin [31]. Salinomycin and other ionophores have shown efficacy in drug-resistant cancers by exploiting metabolic vulnerabilities [27]. Prior work on ionophores in cancer provides a roadmap for optimizing these properties through structural modifications or combination regimens [35]. For example, combining nigericin with BCL-2 inhibitors could synergistically target mitochondrial apoptosis pathways, as suggested by recent preclinical studies in SF3B1-mutant AML [32].

While this study establishes the anti-leukemic potential of nigericin in AML, it has certain limitations. The lack of in vivo validation restricts the translational relevance of the findings. Additionally, validation using primary blasts from FLT3-ITD mutated AML patients is necessary to support results obtained from the MOLM-13 cellular model. Future studies should also investigate nigericin in combination with standard-of-care therapies to evaluate its potential clinical applicability.

## Conclusion

This study demonstrates that nigericin induces apoptosis in AML cells through mitochondrial dysfunction and oxidative stress. These findings contribute to the exploration of mitochondrial-targeted strategies in AML and support further preclinical studies to evaluate the broader applicability and safety profile of nigericin in diverse models of the disease

## Data Availability

All data are available from the corresponding author upon reasonable request.

## Appendix A

**Table A1:** Percentage growth of different cell lines at different drug concentrations (10^−4^, 10^−5^, 10^−6^, and 10^−7^ M) of Nigericin and Doxorubicin

Cancer type	Cell line	Nigericin				Doxorubicin			
		10^−7^ M	10^−6^ M	10^−5^ M	10^−4^ M	10^−7^ M	10^−6^ M	10^−5^ M	10^−4^ M
Human myeloid leukaemia	U-937	65.2	4.2	−17.0	−13.4	34.5	−0.2	2.7	−0.7
Human breast cancer cell	MDA-MB-468	67.3	37.2	−40.5	−78.2	45.4	25.7	−43.2	−73.2
Human lung cancer	HOP-62	74.2	40.3	−27.9	−72.0	94.0	59.0	4.2	−37.4
Human oral squamous cell carcinoma	SCC-40	−36.5	−35.9	−68.0	−70.0	−67.3	6.7	−71.5	−72.5
Monkey kidney normal cell line	VERO	90.9	52.9	−31.8	−49.9	49.0	12.9	−25.8	−47.8
Human leukaemia	HL-60	6.1	−4.2	5.1	2.7	8.0	12.7	5.5	8.4
Human colon cancer	HT-29	13.9	20.7	3.5	−29.7	14.8	35.3	15.5	6.6
Human colon cancer	COLO-205	14.1	17.2	27.6	18.1	33.0	30.2	15.6	18.6
Human cervical cancer	HeLa	6.3	13.8	−24.1	−60.5	−49.8	−53.1	−63.8	−42.4
Human leukaemia	JURKAT	13.6	−7.6	−6.2	7.1	−1.3	7.9	−7.4	−1.5
Human cervical cancer	SiHa	75.6	76.1	52.1	−19.7	−36.5	−6.7	−43.4	−54.0
Human prostate cancer	DU-145	20.2	34.7	18.3	−64.5	14.3	20.2	−2.7	−14.5
Human Hepatoma	Hep-G2	12.3	98.2	17.4	−28.4	27.4	67.3	52.2	−29.3
Human leukaemia	K-562	57.7	1.9	−25.4	−25.1	39.6	40.4	11.3	5.7
Human breast cancer cell	MCF-7	28.8	19.9	9.7	−54.7	11.1	5.2	−4.3	−28.2
Human lung cancer	A-549	15.1	15.5	−2.7	−56.3	7.1	35.1	12.8	5.5
Human prostate cancer	PC-3	26.0	36.5	22.7	−37.4	2.0	26.4	−18.2	−33.3
Human breast cancer cell	MDA-MB-231	47.2	33.6	−11.5	−43.9	−45.4	−50.2	−53.5	−38.8

## References

[1] Kantarjian H, Kadia T, DiNardo C, Daver N, Borthakur G, Jabbour E, et al. Acute myeloid leukemia: current progress and future directions. Blood Cancer J. 2021;11(2):41. doi:10.1038/s41408-021-00425-3; 33619261 PMC7900255

[2] Emadi A, Law JY. Acute Myeloid Leukemia (AML). [cited 2025 May 18]. Available from: https://www.msdmanuals.com/professional/hematology-and-oncology/leukemias/acute-myeloid-leukemia-aml.

[3] Sheth AI, Althoff MJ, Tolison H, Engel K, Amaya ML, Krug AE, et al. Targeting acute myeloid leukemia stem cells through perturbation of mitochondrial calcium. Cancer Discovery. 2024;14(10):1922–39. doi:10.1101/2023.10.02.560330; 38787341 PMC11452272

[4] Tjahjono E, Daneman MR, Meika B, Revtovich AV, Kirienko NV. Mitochondrial abnormalities as a target of intervention in acute myeloid leukemia. Front Oncol. 2025;14:1532857. doi:10.3389/fonc.2024.1532857; 39902131 PMC11788353

[5] Khorashad JS, Rizzo S, Tonks A. Reactive oxygen species and its role in pathogenesis and resistance to therapy in acute myeloid leukemia. Cancer Drug Resist. 2024;7:5. doi:10.20517/cdr.2023.125; 38434766 PMC10905166

[6] Hole PS, Zabkiewicz J, Munje C, Newton Z, Pearn L, White P, et al. Overproduction of NOX-derived ROS in AML promotes proliferation and is associated with defective oxidative stress signaling. Blood. 2013;122(19):3322–30. doi:10.1182/blood-2013-04-491944; 24089327

[7] Lagadinou ED, Sach A, Callahan K, Rossi RM, Neering SJ, Minhajuddin M, et al. BCL-2 Inhibition targets oxidative phosphorylation and selectively eradicates quiescent human leukemia stem cells. Cell Stem Cell. 2013;12(3):329–41. doi:10.1016/j.stem.2012.12.013; 23333149 PMC3595363

[8] Lee JB, Khan DH, Hurren R, Xu M, Na Y, Kang H, et al. Venetoclax enhances T cell-mediated antileukemic activity by increasing ROS production. Blood. 2021;138(3):234–45. doi:10.1182/blood.2023019985; 34292323 PMC8310428

[9] Hole PS, Pearn L, Tonks AJ, James PE, Burnett AK, Darley RL, et al. Ras-induced reactive oxygen species promote growth factor-independent proliferation in human CD34^+^ hematopoietic progenitor cells. Blood. 2010;115(6):1238–46. doi:10.1182/blood-2009-06-222869; 20007804

[10] Trachootham D, Zhou Y, Zhang H, Demizu Y, Chen Z, Pelicano H, et al. Selective killing of oncogenically transformed cells through a ROS-mediated mechanism by β-phenylethyl isothiocyanate. Cancer Cell. 2006;10(3):241–52. doi:10.1016/j.ccr.2006.08.009; 16959615

[11] Huang D, Zhang C, Xiao M, Li X, Chen W, Jiang Y, et al. Redox metabolism maintains the leukemogenic capacity and drug resistance of AML cells. Proc Natl Acad Sci U S A. 2023;120(13):e2210796120. doi:10.1073/pnas.2210796120; 36947513 PMC10068762

[12] Trombetti S, Cesaro E, Catapano R, Sessa R, Lo Bianco A, Izzo P, et al. Oxidative stress and ROS-mediated signaling in leukemia: novel promising perspectives to eradicate chemoresistant cells in myeloid leukemia. Int J Mol Sci. 2021;22(5):2470. doi:10.3390/ijms22052470; 33671113 PMC7957553

[13] Chen Y, Liang Y, Luo X, Hu Q. Oxidative resistance of leukemic stem cells and oxidative damage to hematopoietic stem cells under pro-oxidative therapy. Cell Death Dis. 2020;11(4):291. doi:10.1038/s41419-020-2488-y; 32341354 PMC7184730

[14] Pollyea DA, Stevens BM, Jones CL, Winters A, Pei S, Minhajuddin M, et al. Venetoclax with azacitidine disrupts energy metabolism and targets leukemia stem cells in patients with acute myeloid leukemia. Nature Medicine. 2018;24(12):1859–66. doi:10.1038/s41591-018-0233-1; 30420752 PMC7001730

[15] Pei S, Pollyea DA, Gustafson A, Stevens BM, Minhajuddin M, Fu R, et al. Monocytic subclones confer resistance to venetoclax-based therapy in patients with acute myeloid leukemia. Cancer Discov. 2020;10(4):536–51. doi:10.1158/2159-8290.cd-19-0710; 31974170 PMC7124979

[16] Farge T, Saland E, De Toni F, Aroua N, Hosseini M, Perry R, et al. Chemotherapy-resistant human acute myeloid leukemia cells are not enriched for leukemic stem cells but require oxidative metabolism. Cancer Discov. 2017;7(7):716–35. doi:10.1158/2159-8290.CD-16-0441; 28416471 PMC5501738

[17] Walter RB, Appelbaum FR, Tallman MS, Weiss NS, Larson RA, Estey EH. Shortcomings in the clinical evaluation of new drugs: acute myeloid leukemia as paradigm. Blood. 2010;116(14):2420–8. doi:10.1182/blood-2010-05-285387; 20538802 PMC2953881

[18] Panina SB, Pei J, Kirienko NV. Mitochondrial metabolism as a target for acute myeloid leukemia treatment. Cancer Metab. 2021;9(1):17. doi:10.1186/s40170-021-00253-w; 33883040 PMC8058979

[19] Short NJ, Konopleva M, Kadia TM, Borthakur G, Ravandi F, Dinardo CD, et al. Advances in the treatment of acute myeloid leukemia: new drugs and new challenges. Cancer Discov. 2020;10(4):506–25. doi:10.1158/2159-8290.cd-19-1011; 32014868

[20] Romer-Seibert JS, Meyer SE. Genetic heterogeneity and clonal evolution in acute myeloid leukemia. Curr Opin Hematol. 2021;28(1):64–70. doi:10.1097/MOH.0000000000000626; 33186150 PMC7731048

[21] Arnone M, Konantz M, Hanns P, Paczulla Stanger AM, Bertels S, Godavarthy PS, et al. Acute myeloid leukemia stem cells: the challenges of phenotypic heterogeneity. Cancers. 2020;12(12):3742. doi:10.3390/cancers12123742; 33322769 PMC7764578

[22] Gao G, Liu F, Xu Z, Wan D, Han Y, Kuang Y, et al. Evidence of nigericin as a potential therapeutic candidate for cancers: a review. Biomed Pharmacother. 2021;137:111262. doi:10.1016/j.biopha.2021.111262; 33508621

[23] Deryabin PI, Shatrova AN, Borodkina AV. Targeting multiple homeostasis-maintaining systems by ionophore nigericin is a novel approach for senolysis. Int J Mol Sci. 2022;23(22):14251. doi:10.3390/ijms232214251; 36430735 PMC9693507

[24] Xu Z, Gao G, Liu F, Han Y, Dai C, Wang S, et al. Molecular screening for Nigericin treatment in pancreatic cancer by high-throughput RNA sequencing. Front Oncol. 2020;10:1282. doi:10.3389/fonc.2020.01282; 32850392 PMC7411259

[25] Järås M, Benjamin. Power cut: inhibiting mitochondrial translation to target leukemia. Cancer Cell. 2011;20(5):555–6. doi:10.1016/j.ccr.2011.10.028; 22094249

[26] Birsoy K, Wang T, Chen WW, Freinkman E, Abu-Remaileh M, Sabatini DM. An essential role of the mitochondrial electron transport chain in cell proliferation is to enable aspartate synthesis. Cell. 2015;162(3):540–51. doi:10.1016/j.cell.2015.07.016; 26232224 PMC4522279

[27] Fuchs D, Heinold A, Opelz G, Daniel V, Naujokat C. Salinomycin induces apoptosis and overcomes apoptosis resistance in human cancer cells. Biochem Biophys Res Commun. 2009;390(3):743–9. doi:10.1016/j.bbrc.2009.10.042; 19835841

[28] Kaushik V, Yakisich JS, Kumar A, Azad N, Iyer AKV. Ionophores: potential use as anticancer drugs and chemosensitizers. Cancers. 2018;10(10):360. doi:10.3390/cancers10100360; 30262730 PMC6211070

[29] Yakisich JS, Azad N, Kaushik V, O’Doherty GA, Iyer AKV. Nigericin decreases the viability of multidrug-resistant cancer cells and lung tumorspheres and potentiates the effects of cardiac glycosides. Tumour Biol. 2017 Mar;39(3):101042831769431. doi:10.1177/1010428317694310; 28351327

[30] Goel Y, Yadav S, Pandey SK, Temre MK, Singh VK, Kumar A, et al. Methyl jasmonate cytotoxicity and chemosensitization of t cell lymphoma *in vitro* is facilitated by HK 2, HIF-1α, and Hsp70: implication of altered regulation of cell survival, pH homeostasis, mitochondrial functions. Front Pharmacol. 2021;12:628329. doi:10.3389/fphar.2021.628329; 33716751 PMC7954117

[31] Roulston GDR, Burt CL, Kettyle LMJ, Matchett KB, Keenan HL, Mulgrew NM, et al. Low-dose salinomycin induces anti-leukemic responses in AML and MLL. Oncotarget. 2016;7(45):73448–61. doi:10.18632/oncotarget.11866; 27612428 PMC5341990

[32] Moison C, Gracias D, Schmitt J, Girard S, Spinella J-F, Fortier S, et al. SF3B1 mutations provide genetic vulnerability to copper ionophores in human acute myeloid leukemia. Sci Adv. 2024;10(12):eadl4018. doi:10.1126/sciadv.adl4018; 38517966 PMC10959413

[33] Zhou B, Wang C, Liu X, Wu B, Li J, Yao S, et al. Combination of nigericin with cisplatin enhances the inhibitory effect of cisplatin on epithelial ovarian cancer metastasis by inhibiting slug expression via the Wnt/β-catenin signalling pathway. Oncol Lett. 2021;22(4):700. doi:10.3892/ol.2021.12961; 34457055 PMC8358618

[34] Wu L, Bai S, Huang J, Cui G, Li Q, Wang J, et al. Nigericin boosts anti-tumor immune response via inducing pyroptosis in triple-negative breast cancer. Cancers. 2023;15(12):3221. doi:10.3390/cancers15123221; 37370831 PMC10296105

[35] Sahu AK, Said MS, Hingamire T, Gaur M, Khan A, Shanmugam D, et al. Approach to nigericin derivatives and their therapeutic potential. RSC Advances. 2020;10(70):43085–91. doi:10.1039/d0ra05137c; 35514935 PMC9058090

[36] Yang Z, Xie J, Fang J, Lv M, Yang M, Deng Z, et al. Nigericin exerts anticancer effects through inhibition of the SRC/STAT3/BCL-2 in osteosarcoma. Biochem Pharmacol. 2022;198:114938. doi:10.1016/j.bcp.2022.114938; 35114189

[37] Panina SB, Baran N, Brasil Da Costa FH, Konopleva M, Kirienko NV. A mechanism for increased sensitivity of acute myeloid leukemia to mitotoxic drugs. Cell Death Dis. 2019;10(8):617. doi:10.1038/s41419-019-1851-3; 31409768 PMC6692368

[38] Chen X, Glytsou C, Zhou H, Narang S, Reyna DE, Lopez A, et al. Targeting mitochondrial structure sensitizes acute myeloid leukemia to venetoclax treatment. Cancer Discov. 2019;9(7):890–909. doi:10.1158/2159-8290.CD-19-0117; 31048321 PMC6606342

[39] Rich IN, Worthington-White D, Garden OA, Musk P. Apoptosis of leukemic cells accompanies reduction in intracellular pH after targeted inhibition of the Na+/H+exchanger. Blood. 2000;95(4):1427–34. doi:10.1182/blood.v95.4.1427.004k48_1427_1434. 10666221

[40] Yang Y, An Y, Ren M, Wang H, Bai J, Du W, et al. The mechanisms of action of mitochondrial targeting agents in cancer: inhibiting oxidative phosphorylation and inducing apoptosis. Front Pharmacol. 2023;14:1243613. doi:10.3389/fphar.2023.1243613; 37954849 PMC10635426

